# Role of Artesunate on cardiovascular complications in rats with type 1 diabetes mellitus

**DOI:** 10.1186/s12902-021-00682-0

**Published:** 2021-01-26

**Authors:** Yi Chen, Wei Li, Xiaolin Nong, Chen Liang, Jiaquan Li, Wei Lu, Bingge Wang, Zhong Yuan, Shiying Yang

**Affiliations:** 1 Department of Oral & Maxillofacial Surgery, College of Stomatology, Guangxi Medical University, No.10 Shuangyong Road, Nanning, 530021 Guangxi China; 2 Guangxi Key Laboratory of Oral and Maxillofacial Rehabilitation and Reconstruction, Nanning, 530021 Guangxi China; 3 Guangxi Clinical Research Center for Craniofacial Deformity, Nanning, 530021 Guangxi China; 4 Guangxi Key Laboratory of Oral and Maxillofacial Surgery Disease Treatment, Nanning, 530021 Guangxi China; 5 Medical Science Research Center, Guangxi Medical University, Nanning, 530021 Guangxi China; 6 Life Science Institute, Guangxi Medical University, Nanning, 530021 Guangxi China; 7 School of Information and Management, Guangxi Medical University, Nanning, 530021 Guangxi China

**Keywords:** Artesunate, Type 1 diabetes mellitus, Cardiovascular complication, Glycosylation end product receptor, Nuclear transcription factor

## Abstract

**Background:**

The present study aimed to evaluate the effect of artesunate (ART) on the reduction of cardiovascular complications in a type 1 diabetes model and to investigate the associated mechanism based on the receptor for advanced glycation end-product (RAGE)/NF-κB signaling pathway.

**Methods:**

A total of 40 male Sprague-Dawley rats were randomly divided into five groups: The healthy, diabetic, 50 mg/kg ART (ig) treatment diabetic, 100 mg/kg ART (ig) treatment diabetic, and 6 U/kg insulin (iH) treatment diabetic groups. The treatment lasted 4 weeks after the diabetic model was established via intraperitoneal injection of streptozotocin. Blood samples were collected, and cardiovascular tissues were harvested and processed to measure various parameters after the animals were sacrificed. The myocardium and aortic arch tissues were evaluated using hematoxylin-eosin and Masson staining. Expression levels of RAGE, NF-κB, matrix metalloproteinase MMP9, MMP1 and CD68 in the myocardium and aortic arch tissues were detected using immunohistochemistry, and mRNA expression was determined using reverse transcription-quantitative PCR.

**Results:**

The results of the present study demonstrated that ART treatment may restrain diabetes-induced cardiovascular complications by maintaining heart and body weight while reducing blood glucose, as well as regulating blood lipid indicators to normal level (*P* < 0.05). The expression levels of NF-κB, CD68, MMP1, MMP9 and RAGE were decreased in the ART-treated diabetic rats (*P* < 0.05).

**Conclusions:**

ART treatment may have a protective role against diabetes-associated cardiovascular complications in diabetic rats by inhibiting the expression of proteins in the RAGE/NF-κB signaling pathway and downstream inflammatory factors. High concentrations of ART had a hypoglycemic effect, while a low concentration of ART prevented cardiovascular complications.

## Background

As a systemic disease with multiple organs and tissue damage, diabetes has become the third most common non-infectious disease after cancer and cardiovascular disease. The World Health Organization has predicted that the number of patients with diabetes worldwide will exceed to 300 million by 2025 [1]. Acute and chronic complications of diabetes can jeopardize multiple organs and lead to lesions [2]. Diabetes mellitus-induced cardiovascular complications are a chronic and complex process. Although the mechanisms of diabetes-induced cardiovascular complications have not been unified, the current hypothesis suggests that its mechanism could be associated with the advanced glycation end products (AGEs) signaling pathway [3–5], including the receptor for advanced glycation end-product (RAGE) [6], and effects of inflammatory factors [7, 8], such as macrophage polarization, the macrophage migration inhibitory factor signaling pathway [9], changes in the microvascular wall structure [10], scavenger receptor upregulation [11], insulin resistance [12] and oxidative stress [13].

Recent evidence has suggested that AGEs may activate a series of signaling pathways in combination with their specific receptor RAGE to participate in different stages of the occurrence and development of diabetes and diabetes-induced cardiovascular complications [14, 15]. RAGE is a member of the immunoglobulin superfamily which functions as a transmembrane protein that is widely distributed in the body. RAGE is expressed at a basal level in healthy individuals; however, the expression is increased markedly in pathological conditions, e.g. when an inflammatory reaction causes the cells to be under stress [16]. The combination of AGEs and RAGE induces the production of reactive oxygen species, followed by activation of major cellular signal transduction pathways, such as the NF-κB signaling pathway, resulting in the regulation of a variety of inflammatory and profibrotic factors, such as CD68 and matrix metalloproteinases (MMPs), which cause vascular endothelial damage, cardiomyocyte edema degeneration, myocardial fibrosis and other pathological changes, and are involved in the development of diabetes-induced cardiovascular complications [17–20]. MMPs are closely associated with myocardial fibrosis which may be regulated by RAGE/NF-κB [21–24]. In the present study, the role of the RAGE/NF-κB-mediated signaling pathway in cardiovascular pathology was further explored by constructing a rat model of type 1 diabetes.

Artemisinin is effective in the treatment of malaria. Artesunate (ART) is a water-soluble derivative of Artemisinin, consider to have higher anti-malarial potency, lower toxicity and could be applied intravenously. The chemical formula of ART is C_19_H_28_O_8_. However, in addition to its anti-malarial effect, it has a variety of pharmacological effects, such as anti-fibrosis and antitumor effects [25, 26]. Evidence has suggested that ART has anti-inflammatory effects, and it has been widely used in various inflammation-associated studies. Hou et al [27] demonstrated that ART inhibits the degradation of type II collagen by inhibiting the expression of inflammatory factors, such as CD68, interleukin (IL)-1β and tumor necrosis factor α (TNF-α). It has been reported that ART may inhibit various inflammatory signaling pathways and their downstream factors, such as NF-κB, TNF-α and IL-6 [28–31]. Recently, Li et al [30] suggested that ART has a hypoglycemic activity and demonstrated that ART can inhibit the NF-κB-mediated inflammatory signaling pathway due to its hypoglycemic activity.

To the best of our knowledge, the efficacy and mechanism of action of ART in the treatment of diabetes and its role in cardiovascular complications have not yet been determined. Therefore, the present study aimed to utilize the hypoglycemic activity, anti-inflammatory and anti-fibrotic properties of ART to explore the possibilities of ameliorating diabetes-induced cardiovascular complications by inhibiting the expression of downstream factors of RAGE/NF-κB by acting on the glycosylation pathway.

## Methods

### Ethics statement

The Animal Care & Welfare  Committee of Guangxi Medical University (Nanning, China) approved the experiments, which were performed by the guidelines of the National Institutes of Health (No.201802018). Rats were euthanized by excessive anesthesia with pentobarbital sodium, and all necessary efforts were made to minimize suffering prior to the experiments.

### Experimental animals

A total of 40 adult male Sprague-Dawley rats (weight, 250 ± 20 g) were provided by and kept at the Animal Experimental Center of Guangxi Medical University (Nanning, China). Cages in a specific-pathogen-free level laboratory were used for breeding, and free access to food and water was provided at an ambient temperature of 20–25 °C. The light/dark cycle was 12/12 h, while the daily Illumination time was 12 h.

### Model establishment and grouping

A total of 40 6-week-old Sprague-Dawley rats were randomly divided into five groups as follows: Healthy group (*n* = 8), diabetes mellitus group (DM group; *n* = 8), diabetes mellitus treated with a low dose of ART group (DM + ART 50 mg/kg group; n = 8), diabetes mellitus treated with a high dose of ART group (DM + ART 100 mg/kg group; n = 8) and diabetes mellitus treated with insulin group (DM + INS 6 U/kg group; n = 8). Type 1 diabetes models were established by a single dosage of intraperitoneal injection of streptozotocin (STZ; 60 mg/kg; Sigma-Aldrich; Merck KGaA) dissolved in citrate buffer. Animals in the healthy group were injected intraperitoneally with an equal volume of 0.01 mol/L citrate buffer. Blood samples were collected through the caudal vein to measure the blood glucose levels at the 3rd, 7th, 14th and 28th days after STZ injection. Multiple fasting blood glucose > 16.7 mmol/l was determined to indicate the successful establishment of the diabetes rat model. Subsequently, ART (50 or 100 mg/kg; Guilin Pharmaceutical Co., Ltd.) was administered to the rats in the low- and high-dose ART groups. INS (6 U/kg; Shanghai Fosun Pharmaceutical Co., Ltd.) was administered to rats in the INS group, whereas rats in the healthy and STZ groups were injected with PBS. All treatments were administered once per day for 4 weeks. After the treatment, the rats were euthanized via excessive anesthesia with pentobarbital sodium (150 mg/kg, intraperitoneal injection; Sigma, USA).

### Physiological indices

The body and heart weight, terminal blood glucose, total cholesterol (TC), triglyceride (TG), high-density lipoprotein cholesterol (HDC-C), and low-density lipoprotein cholesterol (LDC-C) levels of each group were measured, and the TC/HDC-C ratio was calculated.

### Hematoxylin and eosin (H&E) and Masson staining

A section (0.5 × 0.5 cm) of the myocardium in the middle part of the left ventricle and aortic arch tissues was removed, fixed in 4% paraformaldehyde solution and embedded in paraffin. The sections were cut into 5-μm-thick sections for H&E and Masson staining. Staining was performed according to the manufacturer’s protocols of the kits. The stained sections were observed under an Olympus-BX41 microscope (original magnification, × 400; Olympus Corporation) and image acquisition was performed.

### Immunohistochemically staining

The sections were dehydrated by gradient ethanol dewaxing, and endogenous peroxidase was blocked with 3% hydrogen peroxide blocking solution after antigen retrieval (the section was immersed in a citrate solution at pH 6.0 for high-pressure repair for 5 min). Subsequently, the sections were blocked in goat serum (R37624, Invitrogen™) for 15 min to reduce non-specific binding of antibodies. After incubation with the primary antibody (RAGE, BS2165; NF-κB, BS4138; MMP9, BS6893; MMP1, BS6232; CD68, BS6885; Bioworld; aortic arch, 1:100; heart, 1:500) overnight at 4 °C, the sections were incubated with horseradish peroxidase-labeled goat anti-rabbit polyclonal antibody for 30 min at 37 °C. Subsequently, the sections were rinsed with PBS, color developed with 3,3′-diaminobenzidine, counterstained with hematoxylin and fixed with a gum solution. For each section, 10 fields were randomly selected for microscopic observation (magnification, 400×) and analyzed using a computer image analysis system (ImageJ; National Institutes of Health). The positive area value of each field of view was calculated and the average was compared among the experimental groups.

### Reverse transcription-quantitative PCR (RT-qPCR)

Total RNA was extracted from aortic arch tissues using TRIzol reagent and transcribed into complementary DNA using the 5X Prime Script RT kit (Takara Bio, Inc.). cDNA was amplified for 60 min at 42 °C, and the terminative reaction temperature was 70 °C for 5 min. qPCR was performed using an ABI PRISM® Step One Plus™ Real-Time PCR system (Applied Biosystems; Thermo Fisher Scientific, Inc.). The amplification conditions were as follows: Holding stage at 95 °C for 30 s, followed by 40 cycles of denaturing at 95 °C for 5 s, annealing at 60 °C for 34 s and a melting curve stage at 60 °C for 60 s. Transcript levels were normalized to those of GAPDH [32] and calculated using the 2^-∆∆Cq^ method. The primer sequences (5′-3′) were as follows: NF-κB p65 forward, AATTTGGCTTCCTTTCTTGGCT and reverse, CTGCGATACCTTAATGACAGCG; RAGE forward, AGGCTGAAGCTGCAGGAATA and reverse, GGGACAGTTGTGAAGGAGGA; MMP9 forward, TGAACGGGAAGCTCACTGG and reverse, TCCACCACCCTGTTGCTGTA; and GAPDH forward, TGAACGGGAAGCTCACTGG and reverse, TCCACCACCCTGTTGCTGTA.

### Statistical analysis

SPSS v22.0 software (IBM Corp.) was used for statistical analysis, and the differences among groups were compared. The measurement data are presented as the mean ± standard deviation (x̄ ± s). The differences among groups were analyzed by one-way ANOVA followed by a Least Significant Difference post hoc test. *P* < 0.05 was considered to indicate a statistically significant difference.

## Results

### Treatment with ART improves the heart and body weight of STZ-induced diabetic rats

**Fig. 1:**
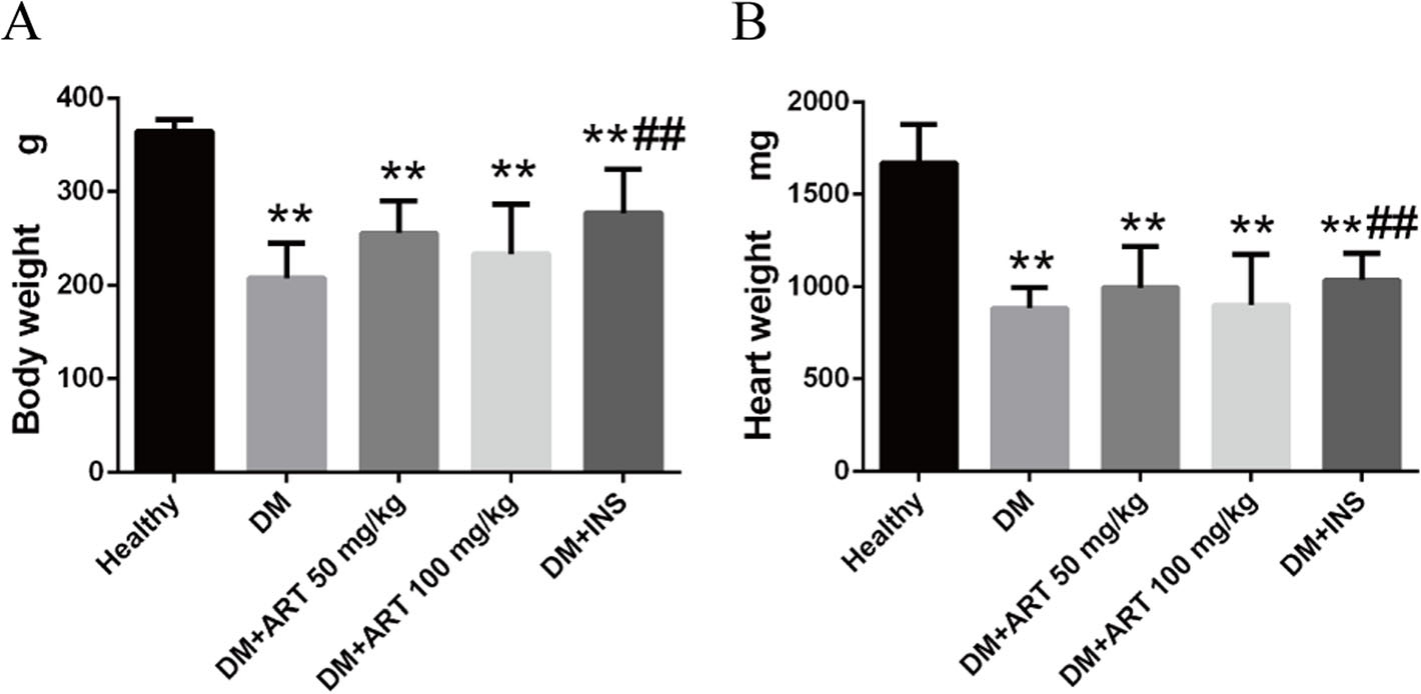
**a** Body and **b** heart weight in different groups. ^**^P < 0.01 vs. healthy group; ^##^*P* < 0.01 vs. DM group. Healthy, control group; DM, type 1 diabetes mellitus mimic model group; DM + ART (50 mg/kg), diabetes mellitus treated with a low concentration of ART; DM + ART (100 mg/kg), diabetes mellitus treated with a high concentration of ART; DM + INS, diabetes mellitus treated with insulin

The heart and body weight of rats in the DM, DM + ART (50 mg/kg), DM + ART (100 mg/kg) and DM + INS groups were decreased compared with those in the healthy group (*P* < 0.01). Compared with the rats in the DM group, the heart and body weight were increased in the DM + ART (50 mg/kg), DM + ART (100 mg/kg) and DM + INS groups (Fig. 1).

### Treatment with ART improves the physiological indices of STZ-induced diabetic rats

**Fig. 2:**
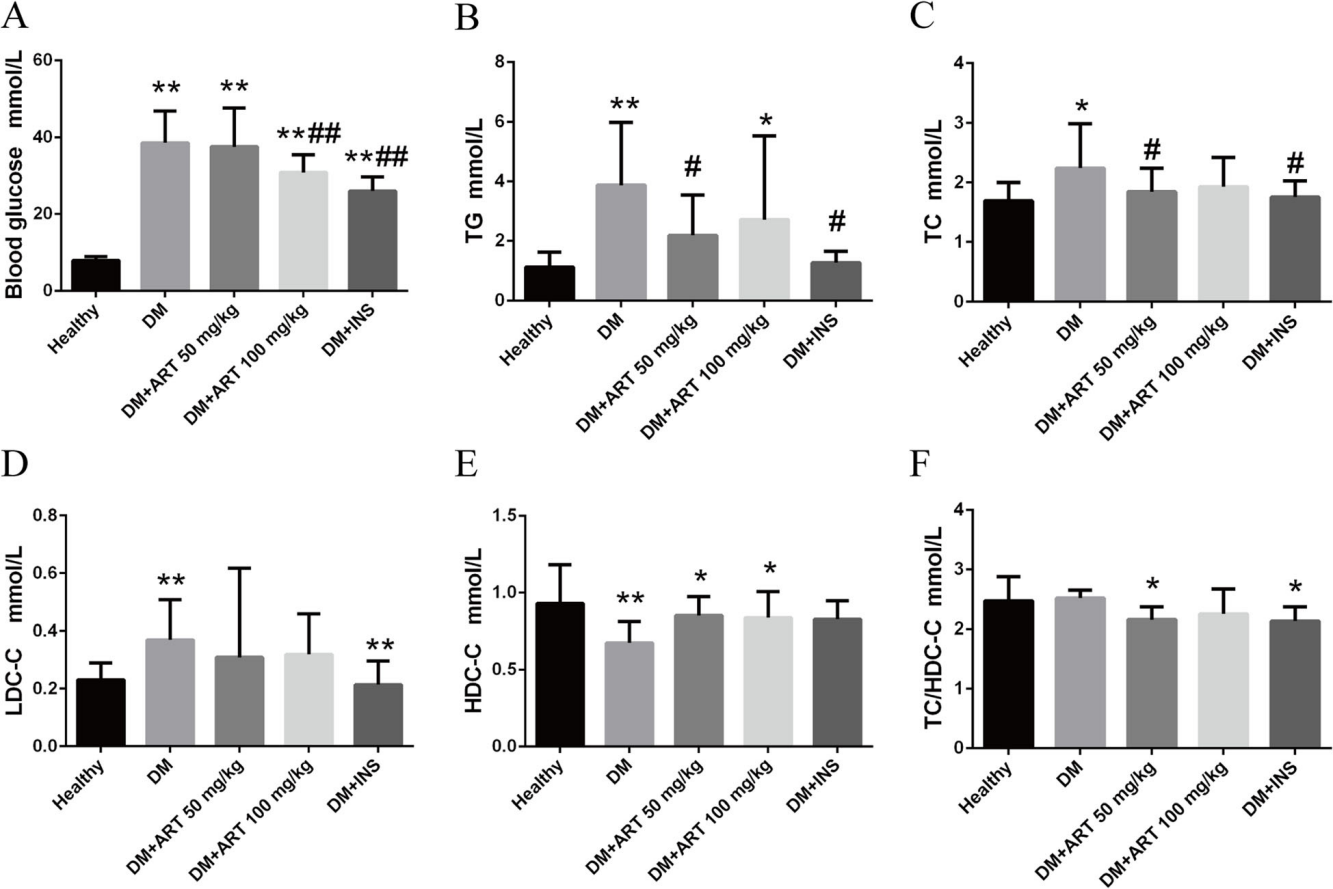
ART improves the physiological indices of STZ-induced diabetic rats. **a** Terminal blood glucose, **b** TG, **c** TC, **d** LDC-C, **e** HDC-C and **f** the ratio of TC/HDC-C in different groups. ^*^P < 0.05 and ^**^P < 0.01 vs. healthy group; ^#^*P* < 0.05 and ^##^*P* < 0.01 vs. DM group. Healthy, control group; DM, type 1 diabetes mellitus mimic model group; DM + ART(50 mg/kg), diabetes mellitus treated with a low concentration of ART; DM + ART (100 mg/kg), diabetes mellitus treated with a high concentration of ART; DM + INS, diabetes mellitus treated with insulin

The plasma terminal blood glucose, TC, TG, LDC-C and the TC/HDC-C ratio levels were increased in the DM, DM + ART (50 mg/kg), DM + ART (100 mg/kg) and DM + INS groups compared with in the healthy group (*P* < 0.05). The concentrations of HDC-C in the DM, DM + ART (50 mg/kg), DM + ART (100 mg/kg) and DM + INS groups were decreased compared with in the healthy group (*P* < 0.01). This indicated an increased risk of coronary atherosclerotic heart disease. Compared with the DM group, plasma blood glucose was decreased in the DM + ART (100 mg/kg) and DM + INS groups (*P* < 0.01). The TC and TG levels were decreased in the DM + ART (50 mg/kg) and DM + INS (6 U) groups compared with in the DM group (*P* < 0.05). There were no significant changes in plasma HDC-C levels and the TC/HDC-C ratio in the other groups compared with in the DM group (Fig. 2).

### Alterations in morphology and fibrosis in the aortic arch and myocardial tissues

**Fig. 3:**
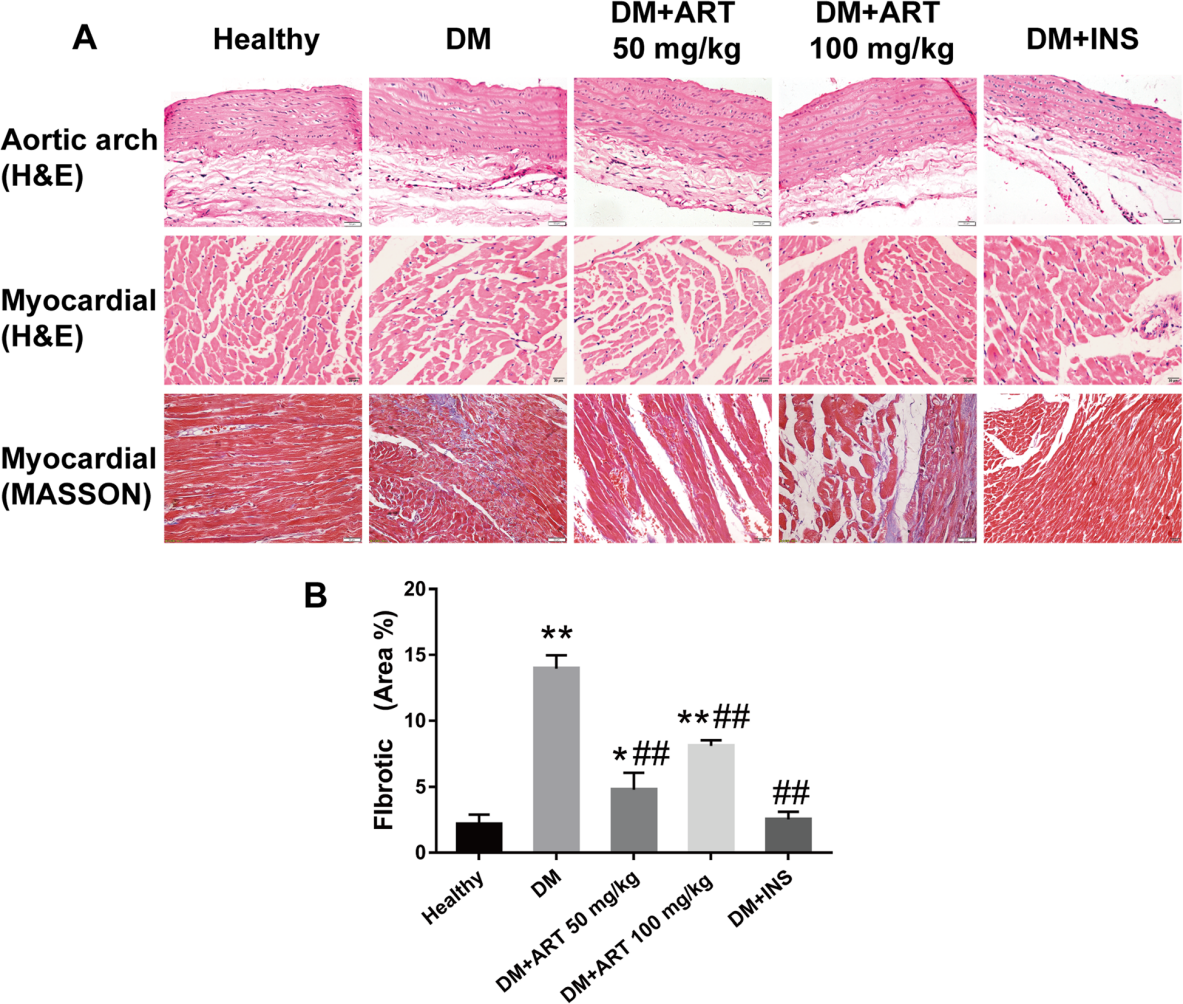
**a** Hematoxylin and eosin staining revealed that the local intima of the aortic arch was markedly thickened in the DM group. The middle layer of elastic fibers was disrupted, arranged in a disorderly manner and there was little inflammatory cell infiltration. The myocardial cell morphology revealed swelling and degeneration, and the myocardial interstitial fiber structure was disordered in the DM groups. Masson staining revealed that the content of myocardial interstitial fibers increased, the morphology was coarse and disordered, and fibers connected into a network in the DM group. The lesions were improved in the medication groups. **b** quantitative analysis of fibrotic in myocardial. *P < 0.05 and **P < 0.01 vs. healthy group; #*P* < 0.05 and ##*P* < 0.01 vs. DM group. Healthy, control group; DM, type 1 diabetes mellitus mimic model group; DM + ART (50 mg/kg), diabetes mellitus treated with a low concentration of ART; DM + ART (100 mg/kg), diabetes mellitus treated with a high concentration of ART; DM + INS, diabetes mellitus treated with insulin

H&E staining revealed that the intima of the aortic arch was smooth and intact in the healthy group. The local intima of the aortic arch was markedly thickened in the DM groups. The middle layer of elastic fibers was disrupted and arranged in a disorderly fashion, and there was little inflammatory cell infiltration. In the DM + ART (50 mg/kg), DM + ART (100 mg/kg) and DM + INS (6 U) groups, a small amount of foam cells was observed, the intimal thickening was markedly reduced, and the elastic fiber structure was complete and arranged neatly. The myocardial cell morphology revealed swelling and degeneration, and the myocardial interstitial fiber structure was disordered in the DM groups. Pathological changes, such as myocardial cell edema, inflammatory cell infiltration and disordered myocardial interstitial fiber arrangement were improved in the DM + ART (50 mg/kg), DM + ART (100 mg/kg) and DM + INS (6 U) groups. Masson staining revealed that the cytoplasm of myocardial cells appeared red and the myocardial interstitial fibers appeared blue. The myocardial interstitial fibers in the healthy group were low in content, complete and arranged in an orderly fashion. In the DM group, the content of myocardial interstitial fibers was increased, the morphology was coarse and disordered, and the fibers were connected into a network. In the DM + ART (50 mg/kg), DM + ART (100 mg/kg) and DM + INS groups, myocardial fibrosis was improved, and the number of collagen fibers was reduced. The myocardial fibrosis changes in the DM + ART (50 mg/kg) group were reduced compared with those in the DM + ART (100 mg/kg) and DM + INS groups (Fig. 3).

### Protein expression levels of RAGE, NF-κB, MMP9, MMP1 and CD68 in myocardial and aortic arch tissues

**Fig. 4:**
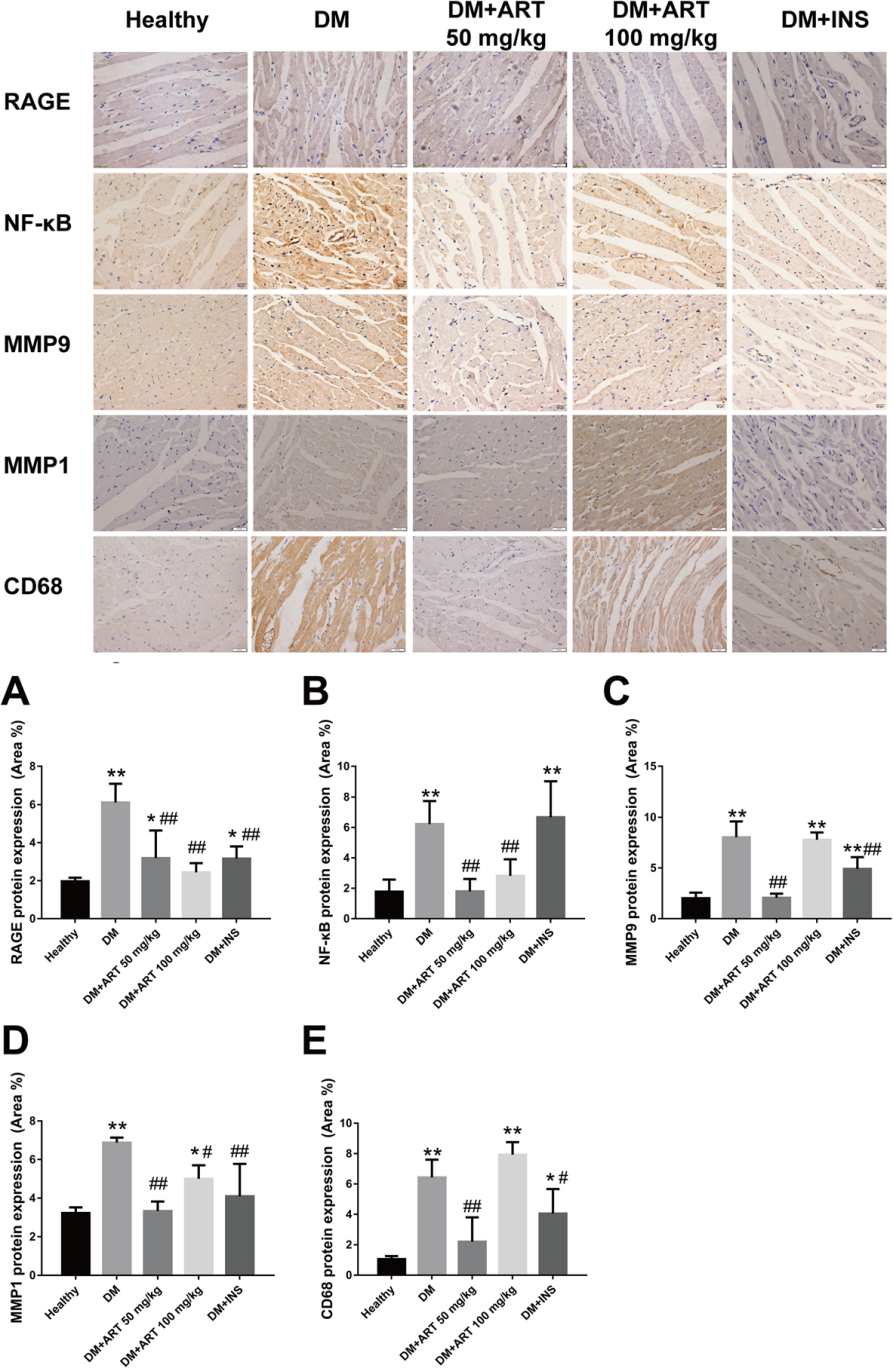
**a** RAGR, **b** NF-κB, **c** MMP9, **d** MMP1 and **e** CD68 protein expression in myocardial tissues. ^*^P < 0.05 and ^**^P < 0.01 vs. healthy group; ^#^*P* < 0.05 and ^##^*P* < 0.01 vs. DM group. Healthy, control group; DM, type 1 diabetes mellitus mimic model group; DM + ART (50 mg/kg), diabetes mellitus treated with a low concentration of ART; DM + ART (100 mg/kg), diabetes mellitus treated with a high concentration of ART; DM + INS, diabetes mellitus treated with insulin

Compared with in myocardial tissues from the healthy group, the protein expression levels of RAGE, NF-κB, MMP9, MMP1 and CD68 were markedly increased in the DM group (*P* < 0.01). The DM + ART (50 mg/kg), DM + ART (100 mg/kg) and DM + INS groups exhibited a marked decrease in RAGE, NF-κB, MMP9, MMP1 and CD68 protein expression. In the DM + ART (100 mg/kg) group, the protein expression levels of RAGE were markedly decreased compared with in the DM + ART (50 mg/kg) group. Conversely, the protein expression levels of NF-κB, MMP9, MMP1 and CD68 were markedly decreased in the DM + ART (50 mg/kg) group (Table 1, Fig. 4).

**Table 1:** RAGR, NF-κB, MMP9, MMP1 and CD68 protein expression levels in myocardial

Group	Mean optical density (MOD)				
	RAGE	NF-κB	MMP9	MMP1	CD68
Healthy	1.972 ± 0.181	1.778 ± 0.793	2.019 ± 0.548	3.233 ± 0.290	1.058 ± 0.192
DM	6.119 ± 0.984^**^	6.219 ± 1.517^**^	8.029 ± 1.563^**^	6.875 ± 0.259^**^	6.425 ± 1.179^**^
DM + ART(50 mg/kg)	3.189 ± 1.451^*##^	1.796 ± 0.820^##^	2.062 ± 0.401^##^	3.330 ± 0.494^##^	2.203 ± 1.602^##^
DM + ART(100 mg/kg)	2.435 ± 0.489^##^	2.809 ± 1.092^##^	7.787 ± 0.713^**^	5.002 ± 0.702^*#^	7.929 ± 0.827^**^
DM + INS	3.165 ± 0.636^*##^	6.673 ± 2.364^**^	4.900 ± 1.164^**##^	4.095 ± 1.682^##^	4.056 ± 1.613^*#^

Results are presented as the mean ± SD. Values of *P < 0.05, **P < 0.01 vs Healthy group. #*P* < 0.05, ##*P* < 0.01 vs DM group indicate a significant difference between each groups

**Fig. 5:**
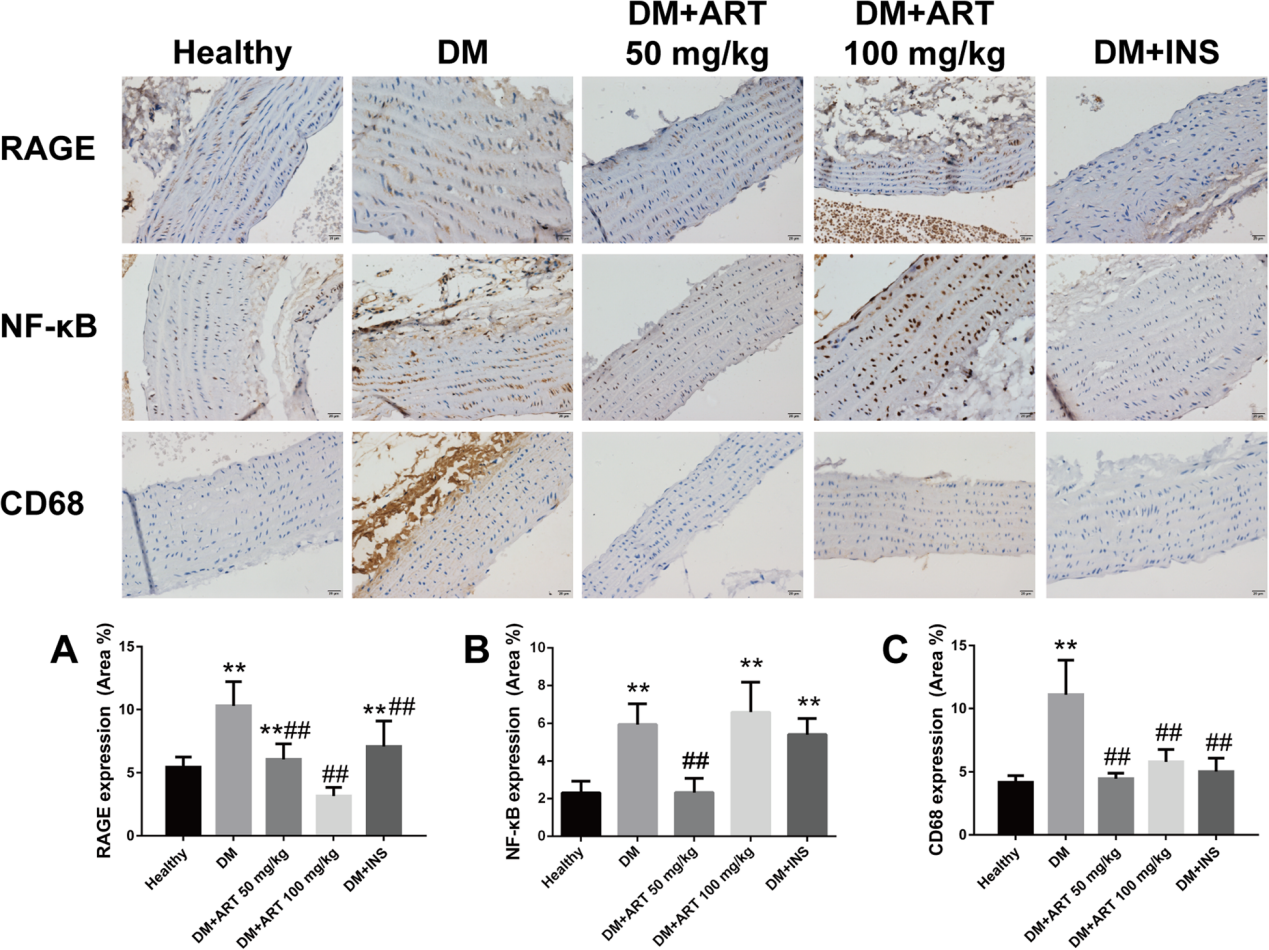
**a** RAGE, **b** NF-κB and **c** CD68 protein expression in aortic arch tissues. ^*^P < 0.05 and ^**^P < 0.01 vs. healthy group; ^#^*P* < 0.05 and ^##^*P* < 0.01 vs. DM group. Healthy, control group; DM, type 1 diabetes mellitus mimic model group; DM + ART (50 mg/kg), diabetes mellitus treated with a low concentration of ART; DM + ART (100 mg/kg), diabetes mellitus treated with a high concentration of ART; DM + INS, diabetes mellitus treated with insulin

In aortic arch tissues from the healthy group, the protein expression levels of RAGE, NF-κB and CD68 were markedly increased compared with those in the DM group (*P* < 0.01). The DM + ART (50 mg/kg), DM + ART (100 mg/kg) and DM + INS groups exhibited marked decreases in RAGE, NF-κB and CD68 protein expression. In the DM + ART (50 mg/kg) group, the protein expression levels of NF-κB and CD68 were markedly decreased compared with those in the DM + ART (100 mg/kg) and DM + INS groups (*P* < 0.01). In the DM + ART (100 mg/kg) group, the protein expression levels of RAGE were markedly decreased compared with those in the DM + ART (50 mg/kg) group (*P* < 0.01; Table 2, Fig. 5).

**Table 2:** RAGE, NF-κB and CD68 protein expression levels in aortic arch

Group	Mean optical density (MOD)		
	RAGE	NF-κB	CD68
Healthy	5.451 ± 0.805	2.312 ± 0.616	4.173 ± 0.517
DM	10.289 ± 1.927**	5.9364 ± 1.087**	11.098 ± 2.744**
DM + ART(50 mg/kg)	6.074 ± 1.227**^##^	2.317 ± 0.751^##^	4.461 ± 0.433^##^
DM + ART(100 mg/kg)	3.146 ± 0.683^##^	6.578 ± 1.601**	5.790 ± 0.994^##^
DM + INS	7.088 ± 2.025**^##^	5.405 ± 0.860**	5.015 ± 1.077^##^

Results are presented as the mean ± SD. Values of *P < 0.05, **P < 0.01 vs Healthy group. #*P* < 0.05, ##*P* < 0.01 vs DM group indicate a significant difference between each groups

### Alterations in aortic arch NF-κB, MMP9 and RAGE gene expression in different groups

**Fig. 6:**
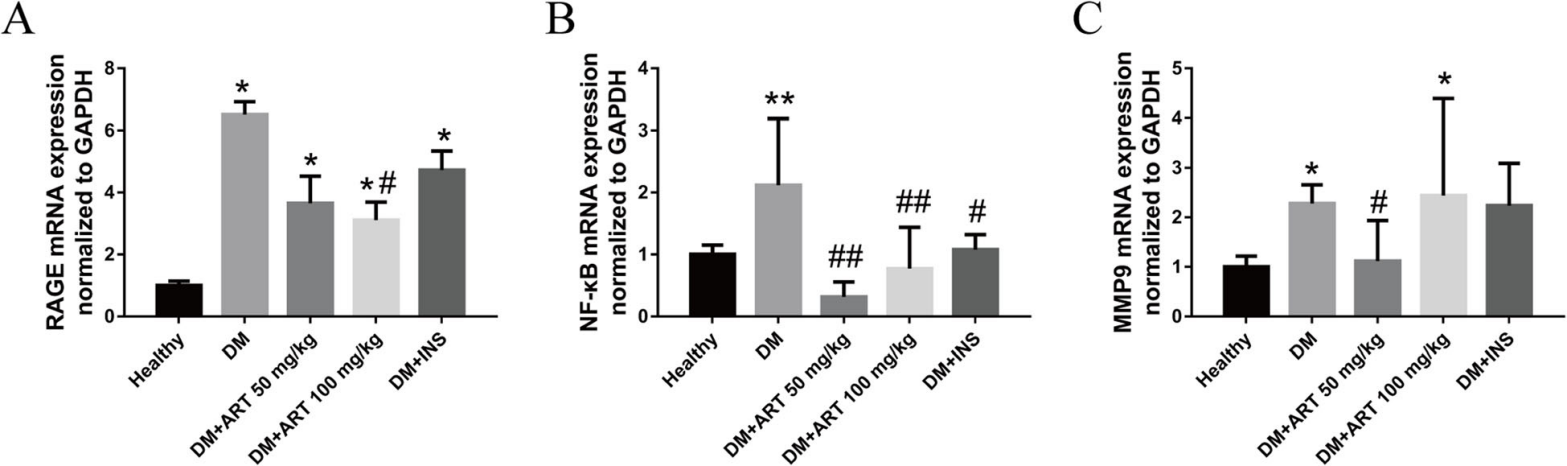
**a** NF-κB, **b** MMP9 and **c** RAGE mRNA expression in aortic arch tissues. ^*^P < 0.05 and ^**^P < 0.01 vs. healthy group; ^#^*P* < 0.05 and ^##^*P* < 0.01 vs. DM group. Healthy, control group; DM, type 1 diabetes mellitus mimic model group; DM + ART (50 mg/kg), diabetes mellitus treated with a low concentration of ART; DM + ART (100 mg/kg), diabetes mellitus treated with a high concentration of ART; DM + INS, diabetes mellitus treated with insulin

The mRNA expression levels of NF-κB, MMP9 and RAGE in the aortic tissues of the DM group were higher than those in the healthy group (*P* < 0.05). The mRNA expression levels of NF-κB, MMP9 and RAGE in the DM + ART (50 mg/kg), DM + ART (100 mg/kg) and DM + INS groups were lower than those in the DM group (*P* < 0.05). Compared with in the DM + ART (100 mg/kg) and DM + INS groups, the mRNA expression levels of NF-κB and MMP9 were markedly decreased in the DM + ART (50 mg/kg) group. However, compared with in the DM + ART (50 mg/kg) and DM + INS groups, the mRNA expression levels of RAGE were markedly decreased in the DM + ART (100 mg/kg) group (Table 3, Fig. 6).

**Table 3:** NF-κB, MMP9 and RAGE mRNA expression levels in aortic arch

Group	The protein expression level(2^-ΔΔCt^)		
	NF-κB	MMP9	RAGE
Healthy	1	1	1
DM	2.119 ± 1.073**	2.277 ± 0.381*	6.580 ± 0.415*
DM + ART(50 mg/kg)	0.315 ± 0.244	1.119 ± 0.817^#^	3.649 ± 0.884*
DM + ART(100 mg/kg)	0.770 ± 0.667^##^	2.440 ± 0.1.953*	3.108 ± 0.585*^#^
DM + INS	1.077 ± 0.245^##^	2.236 ± 0.854	4.728 ± 0.616*

Results are presented as the mean ± SD. Values of *P < 0.05, **P < 0.01 vs Healthy group. #*P* < 0.05, ##*P* < 0.01 vs DM group indicate a significant difference between each groups

## Discussion

ART, as a water-soluble substance of artemisinin, has been widely used in the treatment of malaria in the past. However, the efficacy of ART is not limited to anti-malaria effects; it has certain effects, such as anti-glucose effects [30, 33], anticancer [34], anti-inflammatory and anti-fibrosis [35] effects, as well as inhibiting angiogenesis, which has been strongly substantiated by recent studies. Su et al [36] and Yu et al [29] have suggested that ART can effectively inhibit the NF-κB signaling pathway and serve a corresponding regulatory role in its mediated signaling pathways. The present study aimed to explore whether ART can reverse diabetes and diabetes-induced cardiovascular complications through hypoglycemic activity and anti-inflammation effects by affecting certain targets, such as RAGE and NF-κB. Furthermore, the present study aimed to determine the appropriate dosage of ART treatment.

Existing studies have been mostly directed at type 2 diabetes. The present study established a type 1 diabetes rat model using STZ to explore the cardiovascular pathogenesis of diabetes which avoids the effects of high-sugar and high-fat diets on the cardiovascular structure of animal models. The results demonstrated the intimal thickening of type 1 diabetic rats, vascular endothelial cell injury and myocardial collagen fiber disorder accompanied by increased myocardial interstitial and perivascular fibers, disordered muscle fibers, and increased MMP1/9 protein expression in blood vessels and myocardium. This indicated that cardiovascular fibrosis could be the main pathological process of type 1 diabetes. The mechanism of cardiovascular fibrosis is complex. Previous studies have suggested that the renin-angiotensin-aldosterone system signaling pathway [37], oxidative stress [38], inflammation and other signaling pathways mediate the increase of MMPs and cause fibrosis [39]. The present study mainly addressed the role of RAGE/NF-κB in the pathological process, and the results revealed that the RAGE and NF-κB proteins were highly expressed in association with cardiovascular complications, which demonstrated that they may affect the process to some extent. The blood lipid-related indices of the DM group revealed increased TC, TG and LDC-C levels, and decreased HDC-C levels, suggesting that the incidence of coronary heart disease increased. Compared with the DM group, TC, TG and LDC-C levels were decreased and HDC-C levels were increased following ART treatment, revealing that ART had a hypolipidemic effect. Heart and body weight were decreased in the DM group; however, both of them were decreased after treatment. These observations suggest that ART can improve type 1 diabetes-induced weight loss and cardiac hypertrophy mainly because ART increases the sensitivity of the liver, skeletal muscle and fat cells via the regulation of its glycosylation terminal receptor and nuclear factor protein, which promotes the synthesis of fat. At the same time, it inhibited the myocardial inflammatory signaling pathway and compensated for myocardial ischemia-reperfusion by improving blood lipids, thereby increasing the heart and body weight of rats.

With increasing levels of blood glucose, RAGE expression was markedly increased in the cardiovascular tissues of the DM group. RAGE expression and the blood glucose levels were decreased following treatment with ART which indicated that a high dose of ART had a greater anti-glucose effect than a low dose. ART can be considered to regulate its mediated pathway changes by lowering blood glucose and regulating RAGE expression. In addition, with increased NF-κB, CD68, MMP9 and MMP1 expression in the DM group, NF-κB could mediate the macrophage inflammatory reaction and fibrosis of MMPs. Compared with the DM group, the expression levels of NF-κB, CD68, MMP9 and MMP1 were decreased following ART treatment. By contrast, 50 mg/kg ART treatment had a greater effect than 100 mg/kg ART treatment. It is possible that ART can alter the inflammatory and fibrotic effects in cardiovascular tissues by regulating the NF-κB signaling pathway, and the effect of a low dose could be better. Considering the hepatotoxicity and nephrotoxicity of ART, drug accumulation in the high-dose treatment group may cause abnormal metabolism of the liver and kidney. Toxic substances could be transported to cardiovascular tissues via the blood circulation which has certain adverse effects [40]. Therefore, in the treatment of diabetic cardiovascular complications with ART, the dosage should be considered according to the main therapeutic purpose. The dosage can be increased when it is used to decrease blood glucose, whereas cardiovascular complications should be treated with a low dose.

## Conclusions

ART treatment can improve cardiovascular endothelial cells, cardiomyocyte inflammation and collagen fibrosis. The relative mechanism may include downregulation of the RAGE/NF-κB signaling pathway, and downregulation of CD68, MMP1 and MMP9 protein expression, ultimately affecting inflammatory factors. Surprisingly, we found that high dose artesunate treatment can inhibit the expression of RAGE to play a hypoglycemic effect. Simultaneously, low dose artesunate treatment can down-regulated the mRNA and protein expression of NF-κB, CD68, MMP1 and MMP9 to protect against Cardiovascular injury. And these specific mechanisms need further studies to explore.

## Data Availability

The datasets used and/or analyzed during the current study are available from the corresponding author on reasonable request.

## Abbreviations

ART: Artesunate
H&E: Hematoxylin-eosin staining
HDC-C: High-density lipoprotein cholesterol
IHC: Immunocytochemistry staining
LDC-C: Low-density lipoprotein cholesterol
MMP 1: Matrix Metallopeptidase 1
MMP 9: Matrix Metallopeptidase 9
MOD: Mean optical density
NF-κB: Nuclear transcription factor
PBS: Phosphate buffer saline
RAGE: Glycosylation end product receptor
RQ: Relative quantity
STZ: Streptozocin
T 1 DM: Type 1 Diabetes Mellitus
TC: Total cholesterol
TG: Triglyceride
